# Restriction of SARS-CoV-2 replication by receptor transporter protein 4 (RTP4)

**DOI:** 10.1128/mbio.01090-23

**Published:** 2023-06-29

**Authors:** Ramanjit Kaur, Takuya Tada, Nathaniel Roy Landau

**Affiliations:** 1 Department of Microbiology, NYU Grossman School of Medicine, New York, New York, USA; University of North Carolina at Chapel Hill, Chapel Hill, North Carolina, USA

**Keywords:** coronavirus, RTP4, type-I interferon, SARS-CoV-2, interferons, HCoV-OC43

## Abstract

Severe acute respiratory syndrome coronavirus 2 (SARS-CoV-2) is subject to restriction by several interferon-inducible host proteins. To identify novel factors that limit replication of the virus, we tested a panel of genes that we found were induced by interferon treatment of primary human monocytes by RNA sequencing. Further analysis showed that one of the several candidates genes tested, receptor transporter protein 4 (RTP4), that had previously been shown to restrict flavivirus replication, prevented the replication of the human coronavirus HCoV-OC43. Human RTP4 blocked the replication of SARS-CoV-2 in susceptible ACE2.CHME3 cells and was active against SARS-CoV-2 Omicron variants. The protein prevented the synthesis of viral RNA, resulting in the absence of detectable viral protein synthesis. RTP4 bound the viral genomic RNA and the binding was dependent on the conserved zinc fingers in the amino-terminal domain. Expression of the protein was strongly induced in SARS-CoV-2-infected mice although the mouse homolog was inactive against the virus, suggesting that the protein is active against another virus that remains to be identified.

**IMPORTANCE:**

The rapid spread of a pathogen of human coronavirus (HCoV) family member, severe acute respiratory syndrome coronavirus 2 (SARS-CoV-2), around the world has led to a coronavirus disease 2019 (COVID-19) pandemic. The COVID-19 pandemic spread highlights the need for rapid identification of new broad-spectrum anti-coronavirus drugs and screening of antiviral host factors capable of inhibiting coronavirus infection. In the present work, we identify and characterize receptor transporter protein 4 (RTP4) as a host restriction factor that restricts coronavirus infection. We examined the antiviral role of hRTP4 toward the coronavirus family members including HCoV-OC43, SARS-CoV-2, Omicron BA.1, and BA.2. Molecular and biochemical analysis showed that hRTP4 binds to the viral RNA and targets the replication phase of viral infection and is associated with reduction of nucleocapsid protein. Significant higher levels of ISGs were observed in SARS-CoV-2 mouse model, suggesting the role of RTP4 in innate immune regulation in coronavirus infection. The identification of RTP4 reveals a potential target for therapy against coronavirus infection.

## INTRODUCTION

Severe acute respiratory syndrome coronavirus 2 (SARS-CoV-2) belongs to the *Coronaviridae* family of coronaviruses (CoVs) in the order *Nidovirales*. The viruses are enveloped, single-stranded viruses with a positive-sense RNA genome ~30 kb in length that cause respiratory, hepatic, enteric, and neurological diseases in diverse avian species and a wide range of mammals, including humans (1). CoVs currently in current or recent circulation in human populations include human coronavirus-229E (HCoV-229E), HCoV-OC43, HCoV-HKU1, and HCoV-NL63 and the two emerging CoV strains, severe acute respiratory syndrome coronavirus (SARS-CoV) and Middle East respiratory syndrome coronavirus (MERS-CoV) (2, 3). SARS-CoV-2, the most recently identified HCoV, resulted from the zoonotic transfer from an animal reservoir, resulting in a worldwide pandemic at considerable cost to human health and economic stability (4).

CoVs, like other viruses, are subject to the antiviral effects of the innate immune response. The response consists of a large number of interferon-stimulated genes (ISGs) that interfere with virus replication through a variety of mechanisms (5 - 9). While the viruses encode proteins that dampen interferon (IFN) responses and evade various innate immune mechanisms, it is clear that the innate immune response plays an important role in limiting virus replication and disease pathogenesis, as demonstrated by the increased pathogenicity in patients with mutations in the pathway (10 - 13). CoVs are subjected to restriction from several ISGs. The zinc finger antiviral protein (ZAP) restricts SARS-CoV-2 replication by targeting CpG dinucleotides in viral RNA sequences (14). IFIT1, IFIT3, and IFIT5 with tetratricopeptide repeats inhibit viral protein translation by targeting the 2′-O methyltransferase activity, reducing the proliferation and virulence of SARS-CoV and SARS-CoV-2 (15 - 17). Viperin, an antiviral protein that restricts a broad range of viruses, inhibits the replication of porcine epidemic diarrhea virus, a member of CoV family, by interacting with the viral nucleocapsid (N) protein (18). The bone marrow stromal cell antigen 2 (BST-2) restricts SARS-CoV-2 and HCoV-229E replication by tethering progeny virions to the cellular surface and intracellular membranes, preventing their release (19 - 21). OAS1 was identified in genetic screens and population genetic studies, has been demonstrated to restrict SARS-CoV-2 infection, and plays a role in SARS-CoV-2 pathogenesis (22 - 25). IFN-induced transmembrane proteins (IFITMs) have been shown to both inhibit and enhance the entry of HCoV-OC43 and SARS-CoV-2 (26 - 30). Lymphocyte antigen 6E (LY6E) restricts CoVs block virus entry by interrupting fusion of the viral envelope with the cellular plasma membranes (31 - 33). The IFNγ-inducible lysosomal thiolreductase (GILT) and CD74 have been found to suppress the entry of SARS-CoV-1 into lysosomes (22, 34).

The receptor transporter protein 4 (RTP4) encoded by black flying fox bats, as well as several other bat species, has been shown to be a potent IFN-inducible inhibitor of the replication of Zika virus, West Nile virus, hepatitis C virus, yellow fever virus, and dengue flaviviruses (35, 36). RTP4 orthologs of their natural host were less potent against particular flaviviruses than from nonnatural host species suggesting that the viruses have evolved to escape restriction (35, 36). In humans, RTP4 belongs to a gene family consisting of four members (RTP1, RTP2, RTP3, and RTP4) that serve as chaperones that facilitate the transport of G protein-coupled receptors to the plasma membrane (37 - 39). RTP1 and RTP2 are specifically expressed on olfactory neurons where they are involved in the functional expression of odorant receptors (39 - 42), while RTP3 and RTP4 colocalize with bitter taste receptors (43) and serve to regulate opioid and taste receptors (43). Human RTP4 (hRTP4) is a widely expressed (7, 44, 45) 247 amino acid protein consisting of an amino-terminal (N-terminal) zinc finger domain (ZFD), an intrinsic disordered variable region and a single carboxy-terminal (C-terminal) transmembrane domain (TM). In humans, RTP4 is up-regulated in the endometrium and corpus luteum in early pregnancy (46). Previous studies have shown that bat RTP4 is localized to the Golgi apparatus and the endoplasmic reticulum (41). Bat RTP4 inhibits flavivirus replication by targeting viral double-stranded RNA (ds RNA), preventing genome amplification and virion production (35).

To identify novel ISGs that restrict HCoV replication, we analyzed candidate IFN-inducible genes for their ability to interfere with HCoV-OC43 and SARS-CoV-2 replication. Of the genes that were tested, the most potent inhibitor was hRTP4. The protein prevented viral RNA synthesis and was active against the ancestral Wuhan SARS-CoV-2 as well as the Omicron variants. The protein associated with viral RNA to target the replication phase of viral infection, preventing viral RNA synthesis and the production of virion proteins. Like the bat protein, the N-terminal domain was required for antiviral activity, while the C-terminal was dispensable. RTP4 was induced in SARS-CoV-2-infected K1-hACE2 and K18-hACE2 transgenic mice, but the protein was inactive against the viruses. This study extends the antiviral activity of hRTP4 to a more general inhibitor of both flavivirus and coronavirus replication.

## RESULTS

### hRTP4 inhibits coronavirus replication

**FIG 1:**
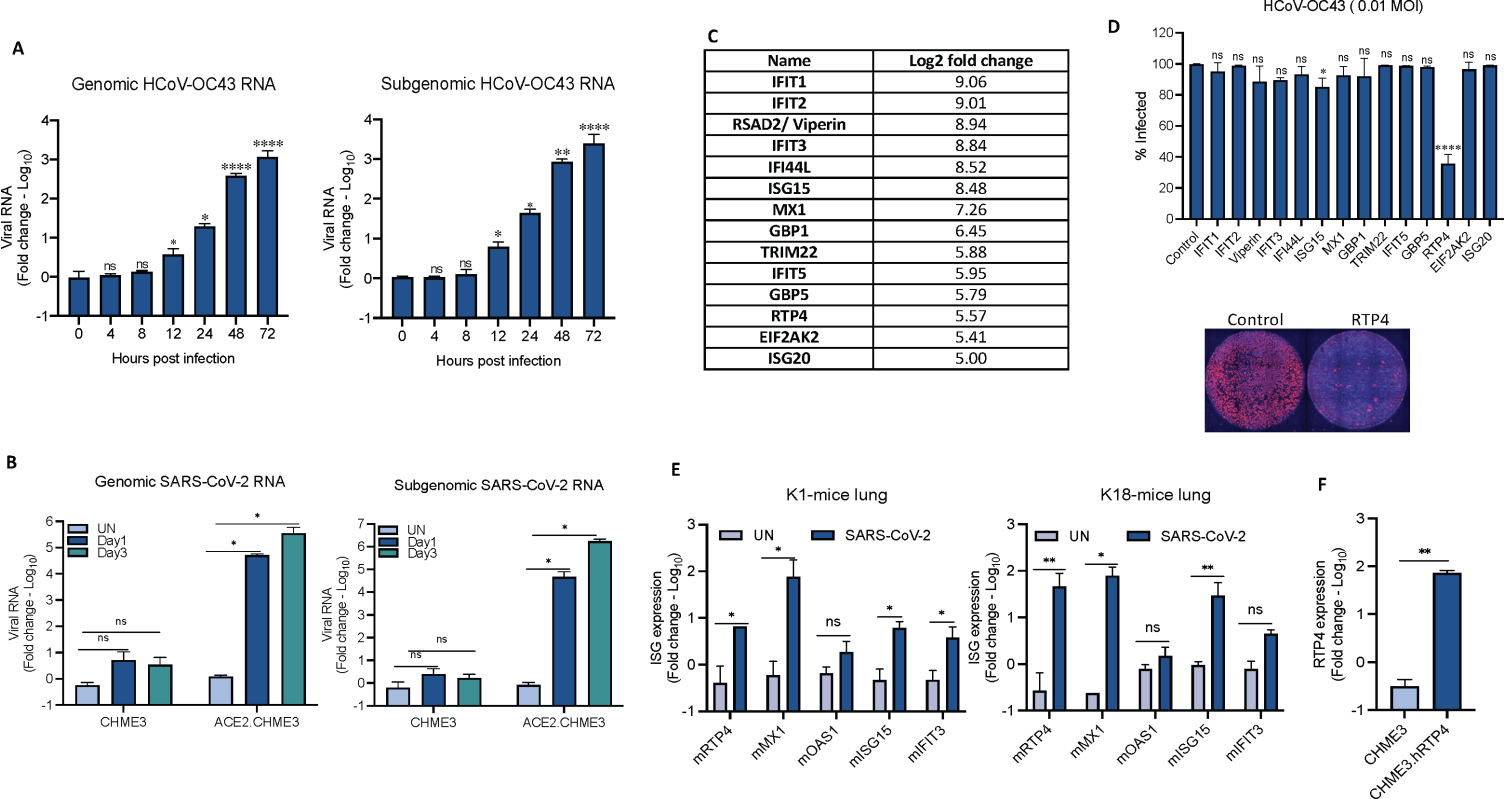
hRTP4 restricts HCoV-OC43 replication. (**A**) CHME3 cells were infected with HCoV-OC43 at an MOI of 0.5. RNA was prepared at the indicated times post-infection, and copy numbers of genomic (left) and subgenomic (right) viral RNA were quantified by qRT-PCR. The data were normalized to GAPDH RNA and expressed as fold-change relative to uninfected cells. (**B**) ACE2.CHME3 cells were infected with SARS-CoV-2 at an MOI of 0.1. At 1 and 3 dpi, RNA was prepared, and genomic (left) and subgenomic (right) RNA copies were quantified by qRT-PCR. The data were normalized to GAPDH and expressed as fold-change relative to the uninfected (UN) cells. All data are the averages of three biological replicates. (**C**) A list of candidate genes identified by RNA-seq that are induced in monocytes more than fivefold upon induction with 100 U IFNα. (**D**) CHME3 cell lines stably expressing candidate ISGs were infected with HCoV-OC43 at an MOI of 0.01. At 48 h post-infection, the cells were fixed and the infected cells in each well were visualized by indirect immunofluorescence for the N protein. High-content microscopy was used to determine the percentage of infected cells as shown in the histogram above with a representative image shown below. Triplicate samples were analyzed and normalized to control HCoV-OC43-infected CHME3 cells. (**E**) K1-hACE2 (left) and K18-hACE2 mice (right) were infected with SARS-CoV-2 WA1/2020 by intranasal instillation or mock uninfected (*n* = 3). At 2 dpi, the mice were killed, and RNA was prepared from the lung tissue. ISG mRNAs were quantified by qRT-PCR with primers hybridizing to mRTP4, mMX1, mOAS1, mISG15, and mIFIT3 mRNA. (**F**) RNA was prepared from CHME3 and CHME3.hRTP4 cell lines. hRTP4 mRNA expression was quantified by qRT-PCR (*P < 0.05, **P < 0.01, ****P < 0.0001).

To study host factors that restrict the replication of beta-CoVs, we tested two family members: the pandemic COVID-19 virus SARS-CoV-2 and the seasonal cold virus HCoV-OC43. For these studies, we tested several cell lines (CHME3, 293T, Hela, MRC5, BHK21, A549, Huh7, and Vero) to identify one that would support the replication of both viruses. Of the cell lines tested, only MRC5 and the human microglial cell-line CHME3 supported HCoV-OC43 (not shown). However, neither expressed the SARS-CoV-2 receptor ACE2 or supported SARS-CoV-2 replication. In light of the favorable growth characteristics of CHME3, we established a clonal CHME3 cell line, ACE2.CHME3, by lentiviral vector transduction that expressed high levels of ACE2 (Fig. S1). To further evaluate the ability of CHME3 cells to support HCoV-OC43 replication, we infected the cells at a multiplicity of infection (MOI) of 0.5 and over the next 72 h, quantified the viral genomic and subgenomic RNA. Genomic viral RNA was first detected 12 h post-infection and increased until 72 h after which the cells died. Similar results were obtained for the subgenomic RNA which is a measure of active virus replication (Fig. 1A). To test the ability of the cells to support SARS-CoV-2 replication, CHME3 and ACE2.CHME3 cells were infected with SARS-CoV-2 WA1/2020 at an MOI of 0.1 and quantified the genomic and subgenomic RNAs. CHME3 cells did not support virus replication, while in the ACE2.CHME3 cells, viral genomic and subgenomic RNAs rose to high levels at 1 day post-infection (dpi) and increased another 10-fold 3 dpi (Fig. 1B).

To identify host factors that restrict CoV replication, we generated a list of genes induced by type-I IFN in primary human monocytes. Monocytes were chosen because they are highly sensitive to type-I IFN and might induce genes that are not typically induced by type-I IFN in transformed cell lines. Monocytes from three donors were treated for 24 h with type-I IFN or were mock treated. RNA was then prepared and subjected to analysis by RNA-seq (47). In order to analyze antiviral activity of ISGs, 14 genes with log_2_-fold change (log2FC) >5 were selected, most of which had previous associations with antiviral activity and encompassed ISGs with previously characterized broad-acting antiviral activities (Fig. 1C; Table S1) (7, 19, 48). We established stable CHME3 cell lines by lentiviral vector transduction for these (RTP4, IFI44L, ISG15, ISG20, MX1, TRIM22, EIF2AK2, IFIT1, IFIT2, IFIT3, IFIT5, GBP1, GBP5, and viperin) (47). To test for restriction of CoV replication by the candidate genes, we infected the CHME3 stable cell lines with HCoV-OC43 and analyzed virus replication 2 dpi by indirect immunofluorescence for the viral N protein using high-content microscope imaging. The results showed that the cell line expressing hRTP4 restricted the replication of the virus (Fig. 1D), while the other 13 genes had no effect on virus replication. The hRTP4 expressing CHME3 cells were not completely protected as 30% of the cells remained infected; however, as the cell lines are polyclonal, they express the proteins at heterogeneous levels which would allow for the infection of some of the cells. Small effects were noted for viperin, ISG15, and IFIT3 but these were not statistically significant.

Our RNA-seq analysis showed that hRTP4 is IFN-inducible in human cells (47). Recent studies have shown SARS-CoV-2 infection of macrophages *in vivo* and *ex vivo* (49 - 51). To determine whether CoV infection would induce RTP4 expression in mice, we infected K1 mice that express human ACE2 from a keratin promoter and K18 mice that express ACE2 from the endogenous promoter with WA1/2020 SARS-CoV-2 and measured the relative abundance of murine RTP4 (mRTP4) and several ISG mRNA transcripts in the lungs. The results showed that mRTP4 mRNA transcripts were strongly induced by SARS-CoV-2 infection in K1-hACE2 and K18-hACE2 transgenic mice (Fig. 1E). The gene was induced more strongly in K18 mice (about 100-fold) a level that was similar to highly inducible mMX1. The induction was about 10-fold in K1 mice which support lower levels of virus replication. mRTP4 was induced to a level greater than those of well-known ISGs mOAS1, mISG15, and mIFIT3. The mRTP4 mRNA copy number in the infected mouse lung cells was comparable to that in the CHME3.hRTP4 cell line, suggesting that the cell line expressed a physiological level of hRTP4 mRNA transcript (Fig. 1F).

**FIG 2:**
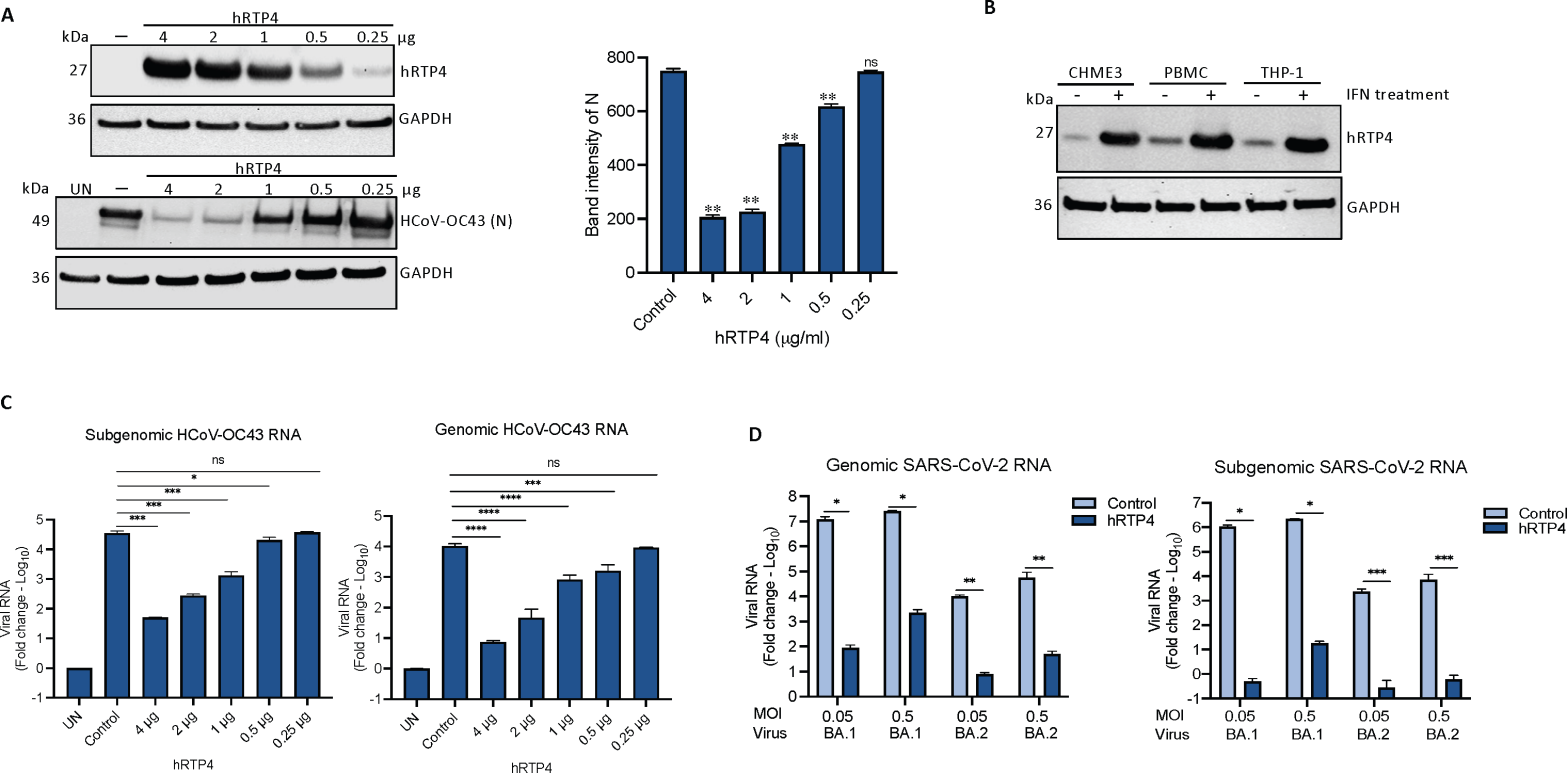
hRTP4 blocks viral RNA and protein production. (**A**) CHME3 cells were transfected with decreasing amounts of hRTP4-2F (4, 2, 1, 0.5, 0.25 µg) along with empty vector control (4 µg) and the next day infected with HCoV-OC43 at an MOI of 0.5. hRTP4-2F was quantified on an immunoblot probed with anti-FLAG antibody and anti-GAPDH antibody as a loading control. The viral N protein in the infected cells was quantified by immunoblot analysis. Band intensities from the blot are graphed (right). (**B**) CHME3, PBMCs, and THP-1 cell lines were treated with 1,000 U/mL of type-I IFN along with untreated controls. After 48 h, hRTP4 protein expression was quantified by immunoblot analysis probed with an anti-RTP4 antibody and anti-GAPDH as a loading control. (**C**) Viral genomic (right) and subgenomic (left) RNAs were quantified by qRT-PCR with transcript-specific primers and normalized to GAPDH. The data are expressed as fold-change relative to the uninfected cells and plotted as the means ± SD and of three biological replicates. The results are representative of two or three experiments. (**D**) ACE2.CHME3 and ACE2.CHME3.hRTP4 cells were infected at an MOIs of 0.05 and 0.5 with Omicron BA.1 and BA.2. At 2 dpi, viral genomic (left) and subgenomic RNAs (right) were quantified by qRT-PCR. The data are normalized to GAPDH and expressed as fold-change relative to the uninfected cells. All data are the averages of three biological replicates (*P < 0.05, **P < 0.01, ***P < 0.001, ****P < 0.0001).

To determine the potency of hRTP4 antiviral activity, we transfected CHME3 cells with a range (0.25–4 µg) of 2X-FLAG-tagged hRTP4 (hRTP4-2F) expression vector or with (0.25–4 µg) of empty vector. Relative hRTP4 protein levels in the transfected cells were determined on an immunoblot probed with anti-FLAG antibody (Fig. 2A). The transfected CHME3 cells were then infected with HCoV-OC43 and virus replication was measured 48 h post-infection by immunoblot analysis of cell lysates for the HCoV-OC43 N protein and by quantification of the viral genomic and subgenomic RNAs by qRT-PCR. The analysis showed a 70% reduction in N protein with 4 and 2 µg pLenti.hRTP4 plasmid and decreased inhibition with lesser amounts of plasmid (Fig. 2A, lower panel) with the band intensities shown in the histogram (Fig. 2A, right). To determine how the level of protein expression in the transfected CHME3 cells compared to those in primary cells expressing physiological levels of RTP4, we analyzed the protein in type-I IFN-treated CHME3, peripheral blood mononuclear cells (PBMCs) and THP-1 cells. The results confirmed the IFN-inducibility of RTP4 in the three cell types and showed that the levels of protein were comparable to those in transfected cells that were sufficient to inhibit virus replication (Fig. 2B).

Analysis of the viral genomic and subgenomic RNA levels showed that 4 µg of hRTP4 plasmid decreased the genomic RNA by about 1,000-fold with a similar decrease in subgenomic viral RNA. As little as 0.5 µg of transfected expression vector plasmid causes a significant decrease in viral RNA (Fig. 2C). To determine whether hRTP4 was active against SARS-CoV-2 variants, we infected the ACE2.CHME3 cells at 0.5 and 0.05 MOI with Omicron variants BA.1 and BA.2 and quantified viral RNA 2 dpi. The results showed that hRTP4 resulted in a >100,000-fold reduction in genomic BA.1 viral RNA at MOI of 0.05 and about 10,000-fold reduction at MOI of 0.5 (Fig. 2D). BA.2 did not replicate to as high titer but the impact of hRTP4 was evident. The effect was even more pronounced for subgenomic RNA, probably because of the absence of input viral RNA copies, unlike for the analysis of the genomic RNA for which input viral RNA is present prior to virus replication.

### hRTP4 restricts coronavirus RNA synthesis

**FIG 3:**
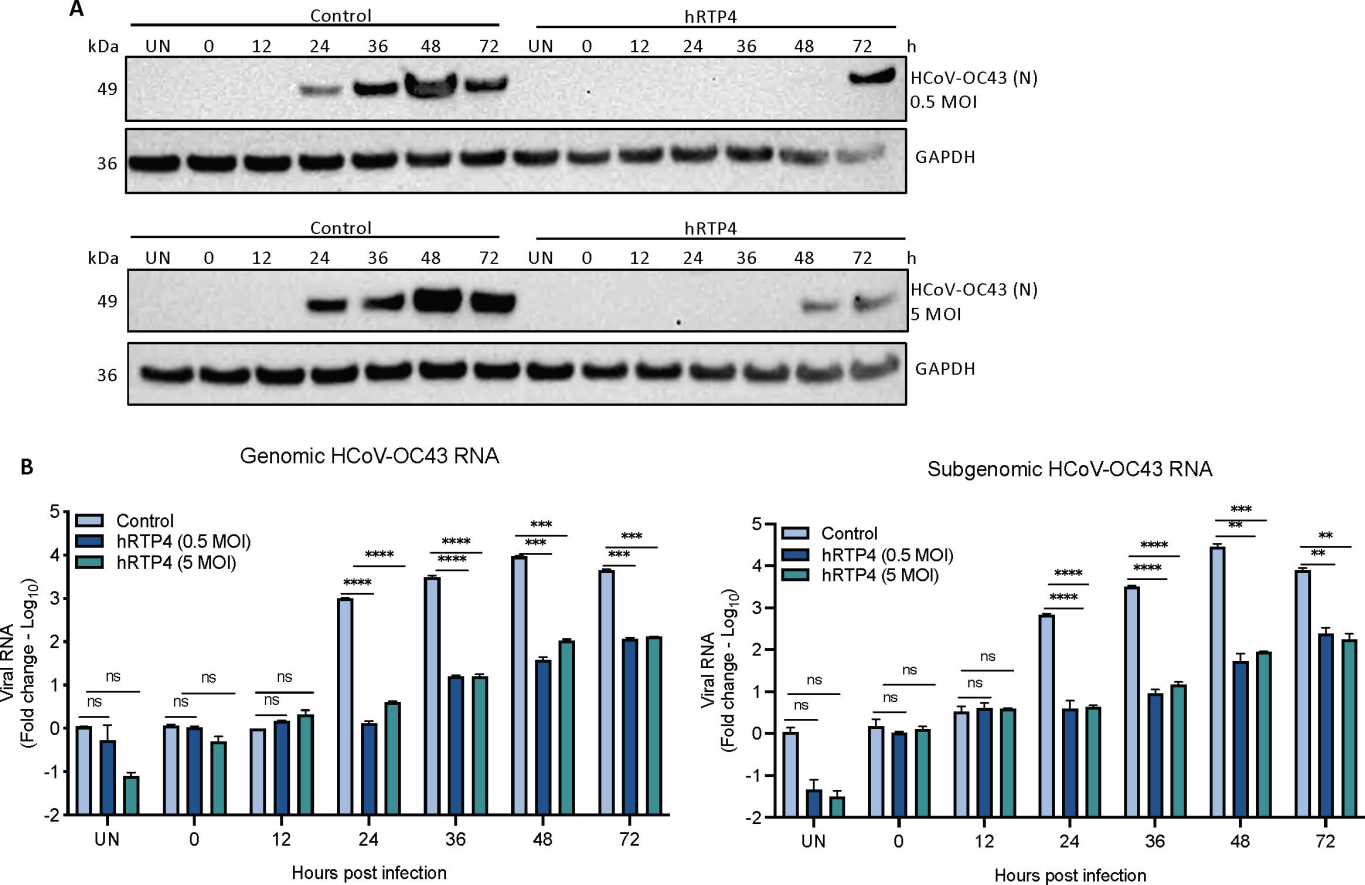
hRTP4 restricts the replication of viral RNA. (**A**) CHME3 cells were transfected with 4 µg of hRTP4-2F expression vector or empty vector and the next day infected with HCoV-OC43 at an MOI of 0.5 (top) or 5 (bottom). Lysates were prepared at 0, 12, 36, 24, 48, and 72 h post-infection and probed for viral N protein and GAPDH. The experiment was repeated twice with similar results. (**B**) RNA was extracted from the infected cells, and the genomic and subgenomic viral RNAs were quantified by RT-PCR. The data are normalized to GAPDH and expressed as a fold-change relative to mock control and plotted as the means ± SD and of three biological replicates (**P < 0.01, ***P < 0.001, ****P < 0.0001).

The CoV replication cycle is initiated by the synthesis of negative-strand RNA using the incoming positive-strand genomic RNA as template. RNA synthesis occurs in the cytoplasm on double-membrane vesicle replication organelles (ROs) that serve as the recruitment site for viral replicative proteins and specific host factors (52). This strategy results in the accumulation of dsRNA and viral N protein as a replicative intermediate within the cytoplasmic viral ROs. The N protein containing ROs in HCoV-OC43-infected cells can be detected with specific antibody. N protein accumulation can be used as a measure of the recruitment of replication complexes that in-turn are related to virus replication initiation. To determine the step in the CoV life cycle that is restricted by hRTP4, HCoV-OC43 replication was examined in CHME3 cells transfected with 4 µg of hRTP4-2F expression vector or empty vector. The transfected CHME3 cells were infected with HCoV-OC43 at MOIs of 0.5 and 5. As a measure of virus spread, accumulation of the HCoV-OC43 N protein was monitored by immunoblot analysis of cell lysates collected over a 3-day period and synthesis of the genomic and subgenomic viral RNAs was quantified by qRT-PCR. Immunoblot analysis showed that in control cells, the N protein appeared at 24 h, increased to its peak level at 48 h and decreased at 72 h at both low and high MOI. In cells expressing hRTP4, there was no detectable N protein until 72 h at low MOI and 48 h at high MOI (Fig. 3A). Analysis of the viral RNAs showed that in the control cells, genomic and subgenomic RNAs became detectable at 24 h, continued to increase slightly at 36 and 48 h, and then decreased slightly at 72 h. The results were similar at both MOIs and for the subgenomic and genomic RNAs (Fig. 3B). In the presence of hRTP4, viral RNA synthesis was suppressed by about 100-fold for both the subgenomic and genomic RNAs at both MOIs although copy numbers increased slightly at 36 and 72 h. The findings suggest that the suppression of N protein synthesis resulted from the inhibition of viral RNA synthesis. We did not rule-out possible effects on virus entry; it is unlikely that RTP4 acts at fusion as it is localized in the endoplasmic reticulum and is not present on the plasma membrane or in endosomes.

### The hRTP4 N-terminal domain is required for antiviral activity

**FIG 4:**
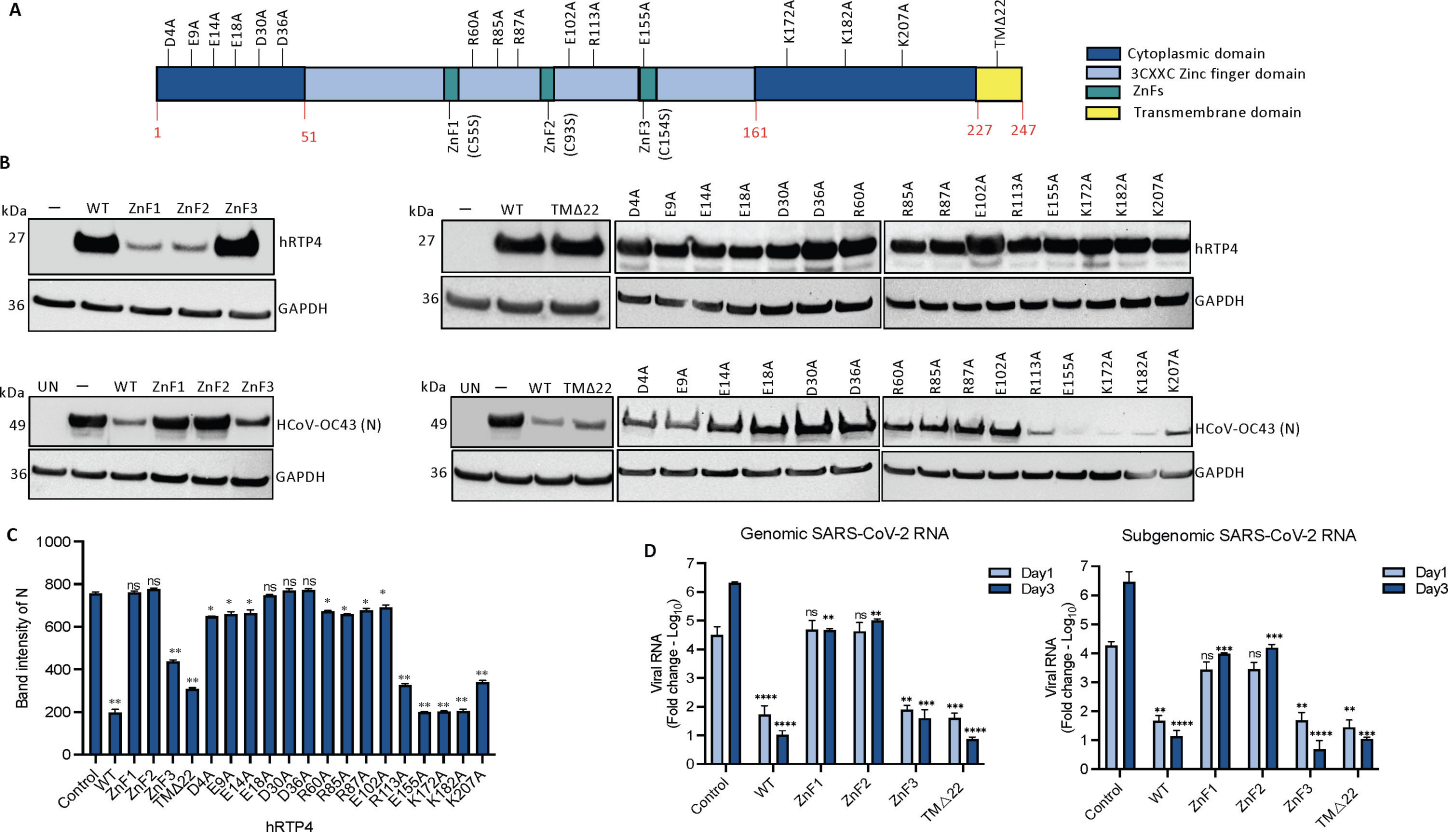
The N-terminal domain of hRTP4 is required for its antiviral activity. (**A**) The domain structure of RTP4 is diagrammed. (**B**) CHME3 cells were transfected with expression vectors for hRTP4 mutants ZnF1, ZnF2, and ZnF3, a 22 amino acid C-terminal deletion TMΔ22 and the indicated charged residue point mutants. Wild-type (WT) hRTP4 and empty vector served as controls. Expression levels were quantified on an immunoblot probed with anti-FLAG and GAPDH antibodies (top). The transfected cells were infected with HCoV-OC43 at an MOI of 0.5. After 48 h, the HCoV-OC43 N protein was quantified by immunoblot analysis with GAPDH as a loading control (bottom). (**C**) Band intensities from the immunoblot analysis for the HCoV-OC43 N protein expression levels are plotted. The experiment was repeated with similar results. All the blots from independent experiments were analyzed at the same exposure time. (**D**) ACE2.CHME3 cells were infected with SARS-CoV-2 at an MOI of 0.1. Viral genomic and subgenomic RNAs were quantified by qRT-PCR at 1 and 3 dpi. The data are normalized to GAPDH and expressed as fold-change relative to the uninfected cells. All data are the averages of three biological replicates (*P < 0.05, **P < 0.01, ***P < 0.001, ****P < 0.0001).

hRTP4 is a 247 amino acid protein consisting of an N-terminal domain with three conserved 3CXXC zinc fingers (ZFD), a central domain containing a variable disordered region and a C-terminal TM anchor (Fig. 4A). The N-terminal 3CXXC ZFD has been shown to be required for antiviral activity against flaviviruses (35). To determine which of the domains are required for antiviral activity, we constructed a series of mutated FLAG-tagged hRTP4 expression vectors. In hRTP4.TMΔ22, the C-terminal TM domain was deleted by truncation of the C-terminal 22 amino acids. The three zinc fingers (ZnFs) were mutated hRTP4.ZnF1-C55S, hRTP4.ZnF2-C93S, and hRTP4.ZnF3-C154S by changing the conserved cysteine to serine. To determine the stability of the mutated and truncated proteins, 293 cells were transfected with each of the vectors or empty vector control and cell lysates were analyzed on an immunoblot probed with anti-FLAG antibody. The results showed that hRTP4.TMΔ22 and hRTP4.ZnF3-C154S were stably expressed. hRTP4.ZnF1-C55S and hRTP4.ZnF2-C93S were expressed only at low level suggesting that zinc fingers 1 and 2 are required for protein stability (Fig. 4A and B, top panel). To measure the antiviral activity of the mutated hRTP4 proteins, CHME3 cells were transfected with the wild-type (WT) and mutated hRTP4 expression vectors and the cells were then infected with HCoV-OC43. At 48 h post-infection, virus replication was measured by immunoblot analysis for the viral N protein. The results showed that the C-terminal truncated TMΔ22 and ZnF3 mutants retained antiviral activity, while ZnF1 and ZnF2 mutants were inactive (Fig. 4B, lower panel, and 4C). The results showed that the C-terminal domain and third zinc finger were dispensable consistent with the findings of Boys et al. (35). Zinc fingers 1 and 2 are required for protein stability and thus their antiviral activity could not be determined.

To further identify critical amino acid residues in the N-terminal and disordered domain, we generated a series mutated expression vectors in which 15 of the charged residues that are conserved in the two domains of human and murine RTP4 were changed to alanine (D4A, E9A, E14A, E18A, D30A, D36A, R60A, R85A, R87A, E102A, R113A E155A, K172A, K182A, and K207A) (Table S2; Fig. S2). Immunoblot analysis of the proteins in transfected 293 cells showed that all were stably expressed (Fig. 4B, lower panel, and 4C). Analysis of their antiviral activity against HCoV-OC43 was tested in transfected CHME3 cells. The results showed that the N-terminal residue mutants (D4A, E9A, E14A, E18A, D30A, D36A, R60A, R85A, R87A, and E102A) were required for antiviral activity whereas those near the C-terminus were not. Mutants toward the end in the N-terminal region and in the middle segment of hRTP4 retained antiviral function (Fig. 4B, lower panel, and 4C).

To determine whether the antiviral mechanism of hRTP4 against SARS-CoV-2 was similar to that of HCoV-OC43, we tested the antiviral activity of the mutated hRTP4s against SARS-CoV-2. For this, we transfected ACE2.CHME3 cells with the mutated expression vectors and then infected them with WA1/2020 SARS-CoV-2. At 1 and 3 dpi, genomic and subgenomic RNA levels were quantified. The results showed that the antiviral activity of the mutants against SARS-CoV-2 mirrored the activity against HCoV-OC43. The C-terminal truncated TMΔ22 and ZnF3 mutants were active, while the N-terminal ZnF1 and ZnF2 hRTP4 mutants partially lost anti-SARS-CoV-2 activity (Fig. 4D). These results suggest that hRTP4 acts by a similar mechanism against both viruses. Together, in transfected CHME3 and ACE2.CHME3 cells, antiviral activity against HCoV-OC43 and SARS-CoV-2 cells required the N-terminal domain, respectively.

### Murine RTP4 is inactive against SARS-CoV-2 and HCoV-OC43

**FIG 5:**
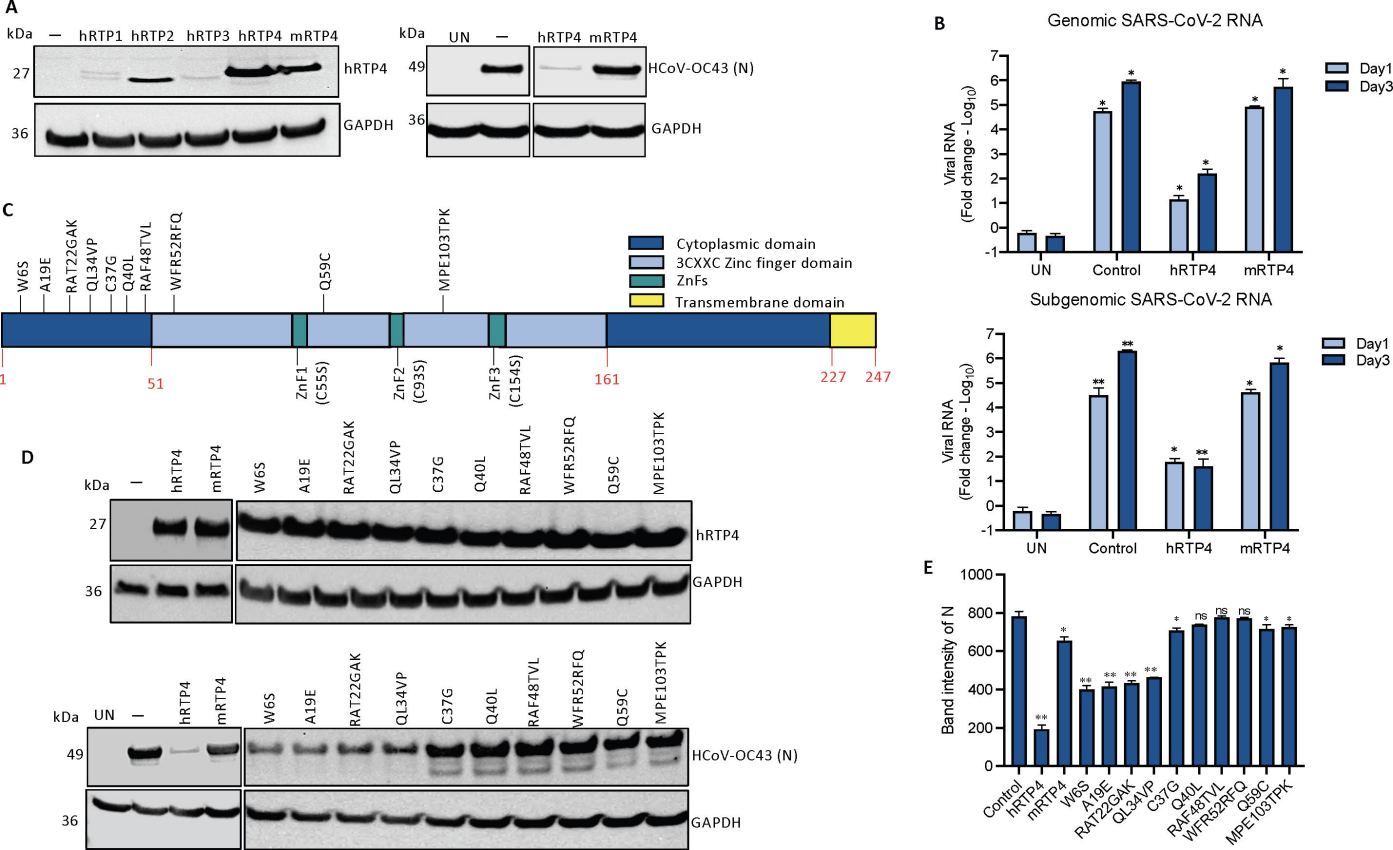
mRTP4 does not restrict coronavirus replication. (**A**) Western blot analysis of lysates from CHME3 cells following transfection of FLAG-tagged RTP4 protein constructs, including hRTP1, hRTP2, hRTP3, hRTP4 (WT), and mRTP4 or empty vector control. The blots were probed with anti-FLAG tag and GAPDH antibodies (left). CHME3 cells expressing the indicated hRTP4 and mRTP4 constructs were infected with HCoV-OC43 at an MOI of 0.5. After, 48 h, the cells were lysed the viral N protein was quantified on immunoblot analysis using GAPDH as a loading control (right). (**B**) ACE2.CHME3 cells were infected with SARS-CoV-2 at an MOI of 0.1 at 1 and 3 dpi. RNA levels were quantified by qRT-PCR. The data were normalized to GAPDH and expressed as the fold-change relative to uninfected cells. The data are averages of three biological replicates. (**C**) Illustration depicting RTP4 human to murine amino acid residue point mutations. (**D**) CHME3 cells were transfected with expression vectors for murine RTP4 and indicated human to mRTP4 residue mutants along with the hRTP4 or empty vector control. Expression levels were quantified on an immunoblot probed with anti-FLAG and GAPDH antibodies (top). The transfected cells were infected with HCoV-OC43 at an MOI of 0.5. After 48 h, the viral N protein was quantified by immunoblot analysis with GAPDH as a loading control (bottom). (**E**) Band intensities from the immunoblot analysis for the HCoV-OC43 N protein expression levels are plotted. The experiment was repeated twice with similar results (*P < 0.05, **P < 0.01).

The RTP family in both mouse and human consists of four members (RTP1, RTP2, RTP3, and RTP4). To determine whether the other family members have activity against CoV replication, we constructed FLAG-tagged expression vectors for the four hRTP4 family members and for mRTP4. The results showed that hRTP1, hRTP2, and hRTP3 were expressed at low to undetectable levels (Fig. 5A) and did not show any antiviral activity (not shown). mRTP4 was expressed at a level similar to that of hRTP4. Analysis of its antiviral activity against HCoV-OC43 in transfected CHME3 cells showed that the protein was inactive against the virus. Analysis of antiviral activity of mRTP4 against SARS-CoV-2 in transfected ACE2.CHME3 cells showed that mRTP4 had no effect at 1 dpi on genomic or subgenomic RNA copy number and a very slight effect, not significantly significant decrease on genomic and subgenomic RNA copy numbers at 3 dpi (Fig. 5B).

To determine the regions and amino acids of mRTP4 that are important for its function, we constructed a series of sequence modifications and point mutations of hRTP4 to substitute amino acids and groups of amino acids with those of the murine protein (Fig. 5C; Fig. S3). We generated 10 mutations in the N-terminal region, which is the domain responsible for antiviral activity (W6S, A19E, RAT22GAK, QL34VP, C37G, Q40L, RAF48TVL, WFR52RFQ, Q59C, and MPE103TPK) based on sequence alignment of the human and murine proteins (Fig. S3; Table S3). Immunoblot analysis of transfected 293 cells showed that the point mutated proteins were stably expressed (Fig. 5D, top panel). To determine the antiviral activity of the mutated proteins, CHME3 cells were transfected with each vector and then infected with HCoV-OC43. Immunoblot analysis for the viral N protein showed that the mutation in the N-terminal domain (W6S, A19E, RAT22GAK, and QL34VP) up to amino acid 34 largely maintained antiviral activity, while those between amino acids 37 and 103 which are close to ZnF1 and ZnF2 were required for antiviral function (Fig. 5D, lower panel, and 5E). The findings show that there are several amino acid positions in the murine protein that render it inactive and that the amino acid sequence of hRTP4 near the zinc fingers cannot be altered without losing antiviral activity.

### hRTP4 binds viral RNA and suppresses the virus replication

**FIG 6:**
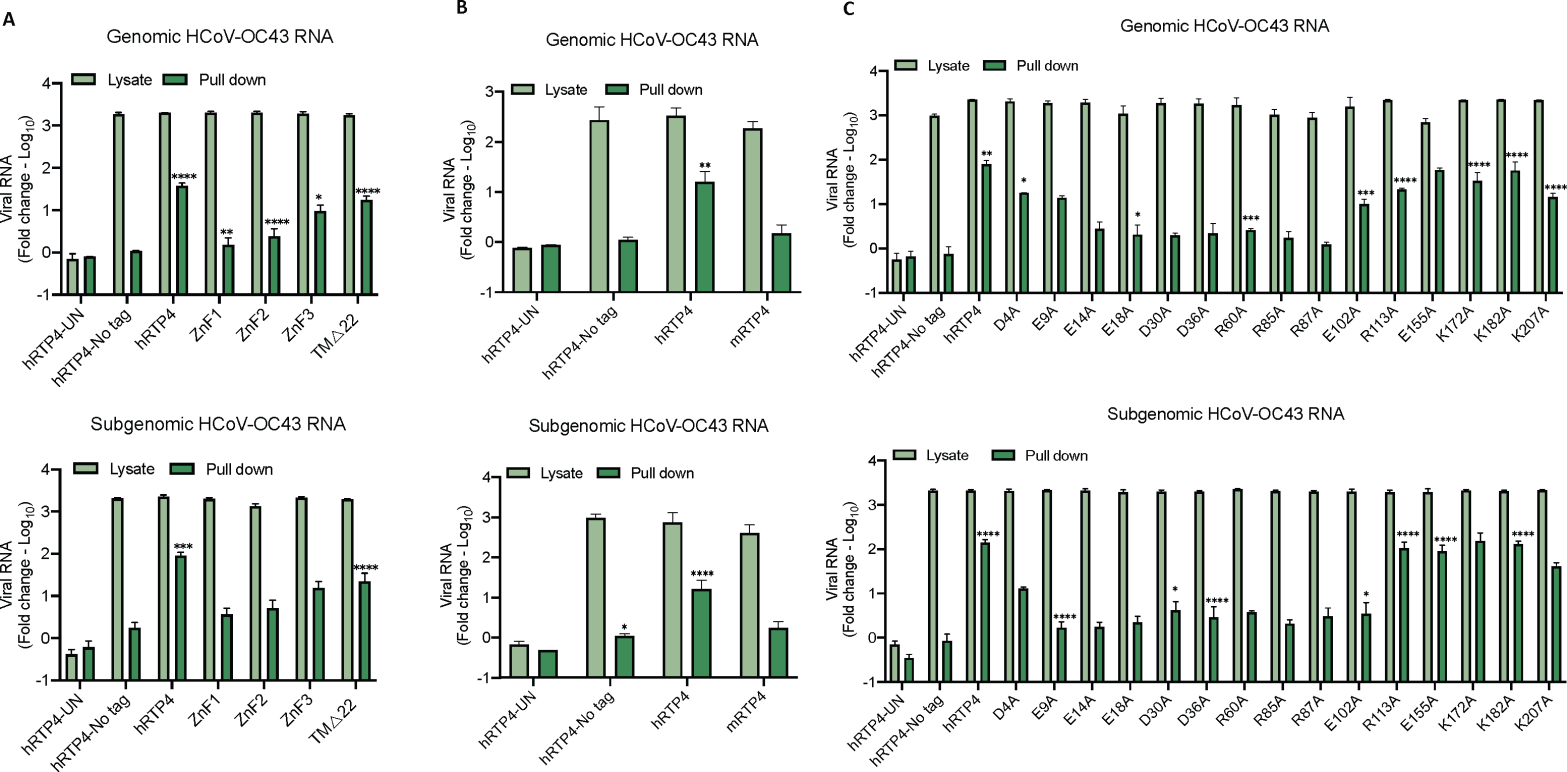
hRTP4 binds to viral RNA. (A through C) CHME3 cells expressing the indicated hRTP4 constructs including (**A**) mutants ZnF1, ZnF2, and ZnF3 and a truncation TMΔ22, (**B**) murine RTP4, and (**C**) hRTP4 charged residues point mutants along with the WT hRTP4 or no FLAG-tag hRTP4 vector control were infected with HCoV-OC43 at an MOI of 5. The lysates were prepared 5 h post-infection and incubated with anti-FLAG M2 magnetic beads. The beads were then pulled-down on a magnetic and the bound virions eluted at low pH. Following elution, RNA was extracted with phenol-chloroform-isoamyl alcohol, precipitated, and analyzed by qRT-PCR for the quantification of bound genomic (top) and subgenomic (bottom) HCoV-OC43 RNAs. The data are expressed as fold-change relative to the uninfected cells. All data are the averages of three biological replicates (*P < 0.05, **P < 0.01, ***P < 0.001, ****P < 0.0001).

Bat RTP4 was shown to interact with the flavivirus genomic RNA (35). To determine whether the human homolog binds to CoV RNA, we tested whether hRTP4 would pull-down the viral RNA. For this, we transfected CHME3 cells with FLAG-tagged hRTP4 expression vector and then infected the cells with HCoV-OC43. The cells were lysed and hRTP4 was pulled-down with anti-FLAG antibody-coated magnetic beads. Unbound complexes were removed and the bound complexes were eluted in low pH buffer. The eluted fractions were phenol extracted and the bound viral RNA was quantified by qRT-PCR. As a specificity control, the pull-down was done with cells expressing an untagged hRTP4. The results showed that the genomic and subgenomic viral RNAs bound to the FLAG-tagged hRTP4. The untagged hRTP4 was not pulled-down, confirming that pull-down was specific (Fig. 6A through C).

To determine the domains of hRTP4 required for viral RNA binding, we tested the ZFD domain mutants and the TMΔ22 carboxy-terminal truncation in the pull-down assay. The results showed that RTP4 with mutations of ZnF1 or ZnF2 bound little viral genomic and subgenomic RNAs. In contrast, the TMΔ22 and ZnF3 hRTP4 proteins retained significant viral RNA binding (Fig. 6A). The murine homolog did not bind the HCoV-OC43 RNA (Fig. 6B). To identify specific amino acids involved in viral RNA binding, we generated a series of charge-to-alanine point mutations throughout hRTP4. Analysis of these in the RNA pull-down assays showed that the mutations from amino acid 9 through 102 (E9A, E14A, E18A, D30A, D36A, R60A, R85A, R87A, and E102A) in the N-terminal region have no or drastically reduced binding with viral RNA, while those in the carboxy-terminus bind to viral RNA with high efficiency (Fig. 6C). The binding to subgenomic RNA was more pronounced than binding to genomic RNA, possibly because the assay was more efficient for smaller RNA. The results show the importance of the charge residues of the protein in the amino terminus, and suggest a role for RNA binding in antiviral activity.

## DISCUSSION

We show here that hRTP4 is a potent inhibitor of coronavirus replication that is active against SARS-CoV-2 and the related coronavirus HCoV-OC43. hRTP4 inhibited the synthesis of HCoV-OC43 viral RNAs resulting in a block to the production of viral proteins. The protein formed complexes with the viral RNA in infected cells and mutated proteins that failed to bind viral RNA did not inhibit virus replication. The N-terminal domain of the protein was required for antiviral activity, while the C-terminal transmembrane domain was dispensable, similar to what was found for flavivirus inhibition by the bat homolog paRTP4 (35). Infection of K1-hACE2 and K18-hACE2 transgenic mice with SARS-CoV-2 induced the expression of mRTP4 yet the murine homolog did not restrict coronavirus replication. These findings extend the role of hRTP4 as a restriction factor that acts on of several classes of RNA viruses.

Boys et al. first identified RTP4 as an ISG with antiviral activity, showing that bat RTP4 was a potent inhibitor of flavivirus replication (35). They suggested that RTP4 could be involved in a Red Queen conflict with flaviviruses, in which diversification of both hosts and viruses has yielded a complex pattern of antiviral specificity of mammalian RTP4 orthologs. In their study, hRTP4 inhibited the Entebbe bat virus replication, a member of flavivirus family but did not appreciably inhibit HCoV-OC43, a finding that differs from ours. The explanation for this difference is not clear but may have been the result of the relatively low level at which the human homolog was expressed in transfected cells (6% that of the bat RTP4). In other studies, hRTP4 was also found to moderately inhibit the replication of norovirus in HG23 cells (53) and yellow fever virus in STAT1-deficient human fibroblasts (7).

Although the murine protein lacked activity against the coronaviruses tested, its expression in mice was induced by SARS-CoV-2 infection, most likely a result of type-I IFN which is present at high levels in SARS-CoV-2 infected mice. The IFN-inducibility of mRTP4 suggests that it has an antiviral role, although the viruses that it targets are not yet determined. Several reports have implicated RTP4 as playing a role in the innate immune response to virus infection (54). The protein was found to be induced in the brains of mice infected with chikungunya and Newcastle disease virus (55, 56). The induction was dependent upon toll-like receptors and the adaptor proteins myeloid differentiation primary response 88 (MyD88) and toll/interleukin-1 receptor domain-containing adaptor inducing IFN-β (TRIF) (55). RTP4 knock-out mice were found to support increased levels of West Nile virus (57). The other RTP family members, RTP1, RTP2, and RTP3, could not be tested for antiviral activity as they were unstable in transfected cells. Their genes were not IFN-inducible and thus not likely to have antiviral function.

In CHME3 cells, hRTP4 decreased the viral genomic and subgenomic HCoV-OC43 RNAs by about 1,000-fold and resulted in the absence of the viral N protein in infected cells. We found that hRTP4 was associated with the viral RNA and the association was mediated by the N-terminal ZFD. These results are consistent with those of Boys et al. who found that bat RTP4 binds the flavivirus dsRNA viral replication intermediate, disrupting the viral replicase complex (35). The association was found to prevent the association of the viral polymerase NS5 and viral helicase NS3, altering binding of the viral polymerase to viral RNA in the replication complex (35). Whether RTP4 has specificity for binding to viral RNAs is not clear.

The findings reported here extend the antiviral activity of RTP4 to another positive-stranded RNA virus family. Its potent activity against SARS-CoV-2 and induction upon infection suggests that it is an important restriction factor that could play a role in disease pathogenesis. Given the profound differences in pathogenicity in COVID-19 severity, the protein could be an important factor contributing to these differences. It does not appear that the virus has a means to avoid the antiviral effects of RTP4. It will be of interest to measure levels of the protein in the cells of SARS-CoV-2-infected individuals and to understand the mechanism by which the protein acts with such broad antiviral activity.

## MATERIALS AND METHODS

### Mice

K1-hACE2 [B6.129S2(Cg)ACE2tm1(ACE2)Dwnt/J] and K18-hACE2 [B6.Cg-Tg(K18-ACE2)2Prlmn/J] transgenic mice were purchased from The Jackson Laboratory and bred in-house. Animal experiments were done under the protocol approved by the NYU Langone Institutional Animal Care and Use Committee (#170304) according to the standards set by the Animal Welfare Act.

### Virus stock preparation

SARS-CoV-2 WA1/2020 P1 virus stock (BEI Resources, NR-52281) was grown on Vero E6 cells by infection at an MOI of 0.01. At 2 h post-infection, input virus was removed and fresh medium was added. After 3 days, the virus-containing supernatant was harvested, filtered through a 0.45 µm filter, and frozen in aliquots at −80°C. Virus titers were determined by plaque assay on Vero E6 cells. The P1 stocks of Omicron BA.1 and BA.2 (BEI Resources, NR-56781) were generated by inoculating ACE2.TMPRSS2.Vero E6 cells at an MOI of 0.1. The P1 stock was expanded by a second round of replication on ACE2.TMPRSS2.Vero E6 cells infected at an MOI of 0.01. After 2 days, the supernatant was collected, filtered, and frozen at −80°C.

MRC5 cells were seeded at a density of 2 × 10^6^ /mL in 100 mm dishes. The following day, the cells were infected with 3 × 10^6^ PFU/mL of HCoV-OC43 (ATCC strain VR-1558) and incubated at 33°C for 4 days at which time 90–100% cells showed a cytopathic effect. The culture supernatant and infected cells were harvested, centrifuged at 1000 × g for 5 minutes, and the supernatant was stored at −80°C. The virus was titered by limiting dilution on MRC5 cells. Infection was scored by cytopathic effect 7 dpi in quadruplicate wells and expressed TCID_50_/mL defined as the dose at which two out of four quadruplicate wells exhibited a cytopathic effect.

Lentiviral vector stock for ACE2 expressing lentiviral vector pLenti.ACE2 was prepared by calcium-phosphate transfection of 293T cells with pLenti.ACE2 (58) and expression plasmids pRSV-Rev, pMDL-X, and VSV-G.

### Mouse infections

Six- to eight-week-old K1-hACE2 and K18-hACE2 transgenic mice were anesthetized with by intraperitoneal injection of ketamine and xylazine and infected intranasally with 2 × 10^4^ PFU SARS-CoV-2 WA1/2020 or the same volume of PBS. At 2 dpi, the mice were killed and the lungs harvested and homogenized in Lysing Matrix D Tubes (MP Biomedicals, Irvine, California, USA) with a FastPrep-24 5G homogenizer (MP Biomedicals). The homogenates were clarified by brief centrifugation and RNA was prepared using a Quick-RNA MiniPrep kit (Zymo Research, Irvine, California, USA).

### Cells

MRC5, CHME3, 293T, and Vero E6 cells were cultured in Dulbecco’s modified Eagle medium (DMEM) supplemented with 10% fetal bovine serum (FBS) and 1% penicillin/streptomycin and incubated at 37°C under 5% CO_2_. To prepare primary human monocytes, PBMCs were purified by Ficoll density gradient centrifugation from Leukopaks provided by the New York Blood Center. Monocytes were purified by plastic adherence and cultured in RPMI containing 10 mM HEPES, 24 µg/mL gentamicin, and 5% heat inactivated pooled human serum. THP-1 cells were cultured in RPMI medium supplemented with 10% FBS and 1% penicillin/streptomycin and incubated at 37°C under 5% CO_2_. To establish ACE2 expressing CHME3 stable cell lines, CHME3 cells were transduced with pLenti.ACE2 vector stock and after 2 days, cloned at limiting dilution in medium containing 1 µg/mL puromycin. The cell clones were stained with anti-ACE2 antibody (NOVUS) and Alexa Fluor 594-conjugated goat anti-mouse IgG antibody (BioLegend, Eugene, Oregon, USA) and screened by flow cytometry to choose a clone that expressed high levels of cell surface ACE2. The data were analyzed with FlowJo software. To establish CHME3 ISG stable cell lines, CHME3 cells were transduced with corresponding ISG lentiviral vector stock and then selected in medium containing 1 µg/mL puromycin and screened by flow cytometry.

### Plasmid construction

hRTP4 expressing lentiviral vector was generated by amplifying an hRTP4 cDNA with a forward primer containing a Spe-I restriction enzyme site and reverse primer containing 2X FLAG tag and Sal-I restriction enzyme site. The amplicon was cleaved with Spe-I and Sal-I and cloned into pLenti6.3/V5-DEST-GFP in place of GFP. hRTP4 TM domain truncation was generated using existing hRTP4 plasmid construct with a forward primer containing a Spe-I restriction enzyme site and reverse primer containing 2X FLAG tag and Sal-I restriction enzyme site. Mutations in the ZFDs were introduced by overlap extension PCR and mutations of the charged residues and the human to murine RTP4 mutants were introduced by quick change site-directed mutagenesis. Expression vectors for hRTP1, hRTP2, hRTP3, and mRTP4 were generated by amplifying the corresponding cDNA sequences (GenScript, Piscataway, New Jersey, USA) with a forward primer containing a Spe-I site and reverse primer containing 2X FLAG tag and Sal-I site and ligating to pLenti6.3/V5-DEST. The sequences of all plasmid constructs were confirmed by sequencing. The primer sequences of the constructs are shown in Table S4.

### HCoV-OC43 viral replication assay

CHME3 cell lines expressing different ISGs were seeded into black 96-well plates at 90% confluency. The next day, the cells were infected with HCoV-OC43 at an MOI of 0.01 and incubated at 33°C. At 48 h post-infection, the cells were fixed with 4% PFA for 20 minutes and permeabilized by treatment for 15 minutes in 0.05% Triton X-100/PBS. The cells were blocked for 1 h in 4% FBS and then stained overnight at 4°C with anti-OC43-N antibody (Sigma-Aldrich, Damstadt, Germany). The antibody was then removed by three washes with PBS, and the cells were stained for 1 h with Alexa Fluor 594 (Invitrogen, Eugene, Oregon, USA) and DAPI at room temperature. Images were analyzed using a Cell Insight CX7 LZR high-content screening platform with HCS Navigator software for DAPI and Alexa Fluor 647.

### Western blotting

Transfected cells were lysed in buffer containing 10 mM Tris HCl, pH 7.5, 150 mM NaCl, 2 mM EDTA, 0.5% NP-40, and protease inhibitor cocktail and lysate protein concentrations were determined. The cell lysates (40 µg) were separated by SDS-PAGE, transferred to polyvinylidene difluoride membranes, and probed with mouse anti-FLAG antibody (Sigma-Aldrich), mouse anti-coronavirus OC43-N antibody (Sigma-Aldrich), rabbit anti-RTP4 antibody (Epigentek, Farmingdale, New York, USA), and anti-GAPDH antibody (Life Technologies, Carlsbad, California, USA) followed by goat anti-mouse HRP-conjugated second antibody (Sigma-Aldrich). The blots were washed and visualized using luminescent HRP substrate (Millipore, Burlington, MA, USA) on an iBright CL1000 imaging system.

### RTP4: RNA complex pull-down

CHME3 cells were transiently transfected with hRTP4-2F expression vector by lipofection with Lipofectamine 2000 (Invitrogen) and then infected with HCoV-OC43 at an MOI of 5. After 2 days, the transfected cells were lysed in NP-40 lysis buffer (10 mM Tris HCl, pH 7.5, 150 mM NaCl, 2 mM EDTA, 0.5% NP-40, and protease inhibitor cocktail). The cell lysates (10 µg) were incubated for 1 h at 4°C with 20 µL anti-FLAG M2 magnetic beads (Sigma-Aldrich). The beads were pulled down in a magnetic separator and washed with buffer containing 50 mM Tris HCl, pH 7.5, and 150 mM NaCl. Bound protein complexes were eluted in buffer containing 0.1 M Glycine HCl, pH 3.0. The eluted proteins were analyzed on an immunoblot probed with mouse anti-FLAG antibody (Sigma-Aldrich) followed by goat anti-mouse HRP-conjugated second antibody (Sigma-Aldrich). RNA that had been pulled down with the RTP4 was isolated by phenol extraction followed by ethanol precipitation. Genomic and subgenomic HCoV-OC43 RNAs that have been pulled down were quantified by qRT-PCR.

### Transfection of 293T cells and gene expression *in vitro*

293T cells were seeded at 2 × 10^5^ cells/well in 6-well plate were transiently transfected with 4 µg of expression vectors along with the empty vector control using Lipofectamine 2000 (Invitrogen). After 2 days, the transfected cells were lysed in NP-40 lysis buffer and the cell lysates were analyzed on an immunoblot probed with mouse anti-FLAG antibody (Sigma-Aldrich) and anti-GAPDH antibody (Life Technologies) followed by goat anti-mouse HRP-conjugated second antibody (Sigma-Aldrich).

### HCoV-OC43 and SARS-CoV-2 infection *in vitro*

CHME3 cells (2 × 10^5^) were transfected with RTP4 expression vectors using Lipofectamine 2000. One dpi, the cells were infected with HCoV-OC43 at an MOI of 0.5 and 5 and incubated at 33°C. The cells were lysed on days 1 and 3 and the lysates were analyzed on an immunoblot probed with mouse anti-coronavirus OC43-N antibody (Sigma-Aldrich) followed by goat anti-mouse HRP-conjugated second antibody (Sigma-Aldrich). The infectivity for experiments was quantified by qRT-PCR. All HCoV-OC43 infections were done at Biosafety Level 2 (BSL2). SARS-CoV-2 (MOI = 0.1), Omicron BA.1, and BA.2 (MOI = 0.05, 0.5) infections in a BSL3 facility according to institutional guidelines provided by the NYU Langone and Institutional Animal Care and Use Committee according to the standards set by the Animal Welfare Act.

### Quantitative RT-PCR

RNA was isolated from virus-infected CHME3 cells using Quick-RNA MiniPrep kit (Zymo Research). qRT-PCR analysis was done with TaqMan Fast Virus 1-Step Master Mix using primers in Orf1ab to amplify genomic HCoV-OC43 RNA and primers in the N gene to amplify subgenomic HCoV-OC43 RNA. SARS-CoV-2 genomic and subgenomic RNA levels were determined in SARS-CoV-2-infected ACE2.CHME3 cells and lung. Primer sequences and probe are detailed in Table S5. Relative RNA copy numbers were calculated by the comparative C_T_ method with GAPDH as the internal control.

### Measurement of ISGs

RNA was prepared from 200 µL homogenized lung using the Quick-RNA MiniPrep kit (Zymo Research). cDNA was reverse-transcribed using Transcriptor RT (Roche, Mannheim, Germany) with random hexamers. qPCR was performed using PowerUp SYBR Green Master Mix (Applied Biosystems, Waltham, Massachusetts, USA) with a typical three-step PCR protocol. The PCR was set for 40 cycles of 95°C/15 s, 60°C/30 s, and 95°C/15 s. Signals were normalized to GAPDH, and quantification of relative gene expression was relative to untreated controls with comparative C_T_ method. Primer sequences are detailed in Table S5.

### Quantification and statistical analysis

Statistical analyses were determined using GraphPad Prism, and statistical significance was determined with the two-tailed, unpaired *t*-test or two-way ANOVA. All the experiments were performed in duplicates or triplicates. Confidence intervals are shown as the mean ± SD or SEM (ns, not significant; *P ≤ 0.05, **P ≤ 0.01, ***P ≤ 0.001, ****P ≤ 0.0001).

## SUPPLEMENTAL MATERIAL

Fig. S1 mbio.01090-23-s0001.tif ACE2 expression levels in ACE2.CHME3 cell line.

Fig. S2 mbio.01090-23-s0002.tif An amino acid sequence of hRTP4 with details on the positions of single-site point mutants, truncation and ZFD mutants.

Fig. S3 mbio.01090-23-s0003.tif An amino acid alignment of hRTP4 and mRTP4 with details on the human to murine RTP4 mutants.

Table S1 mbio.01090-23-s0004.xlsx RNA seq data.

Table S2 mbio.01090-23-s0005.tif Mutagenesis strategy for 15 single-site mutants of hRTP4.

Table S3 mbio.01090-23-s0006.tif Mutagenesis strategy for 10 human to mouse RTP4 residues mutants.

Table S4 mbio.01090-23-s0007.tif Primer sequences of the plasmids constructed in the study.

Table S5 mbio.01090-23-s0008.tif Oligonucleotide sequences for PCR primers and probes used in the study.

Supplemental legends mbio.01090-23-s0009.docx Legends for Fig. S1 to S3 and Tables S1 to S5.

ASM does not own the copyrights to Supplemental Material that may be linked to, or accessed through, an article. The authors have granted ASM a non-exclusive, world-wide license to publish the Supplemental Material files. Please contact the corresponding author directly for reuse.
