# Supplementary figures and images for: Restriction of SARS-CoV-2 replication by receptor transporter protein 4 (RTP4)
Source: mBio. 2023 Jun 29;14(4):e01090-23. doi: 10.1128/mbio.01090-23 (PMC10470548; doi:10.1128/mbio.01090-23)

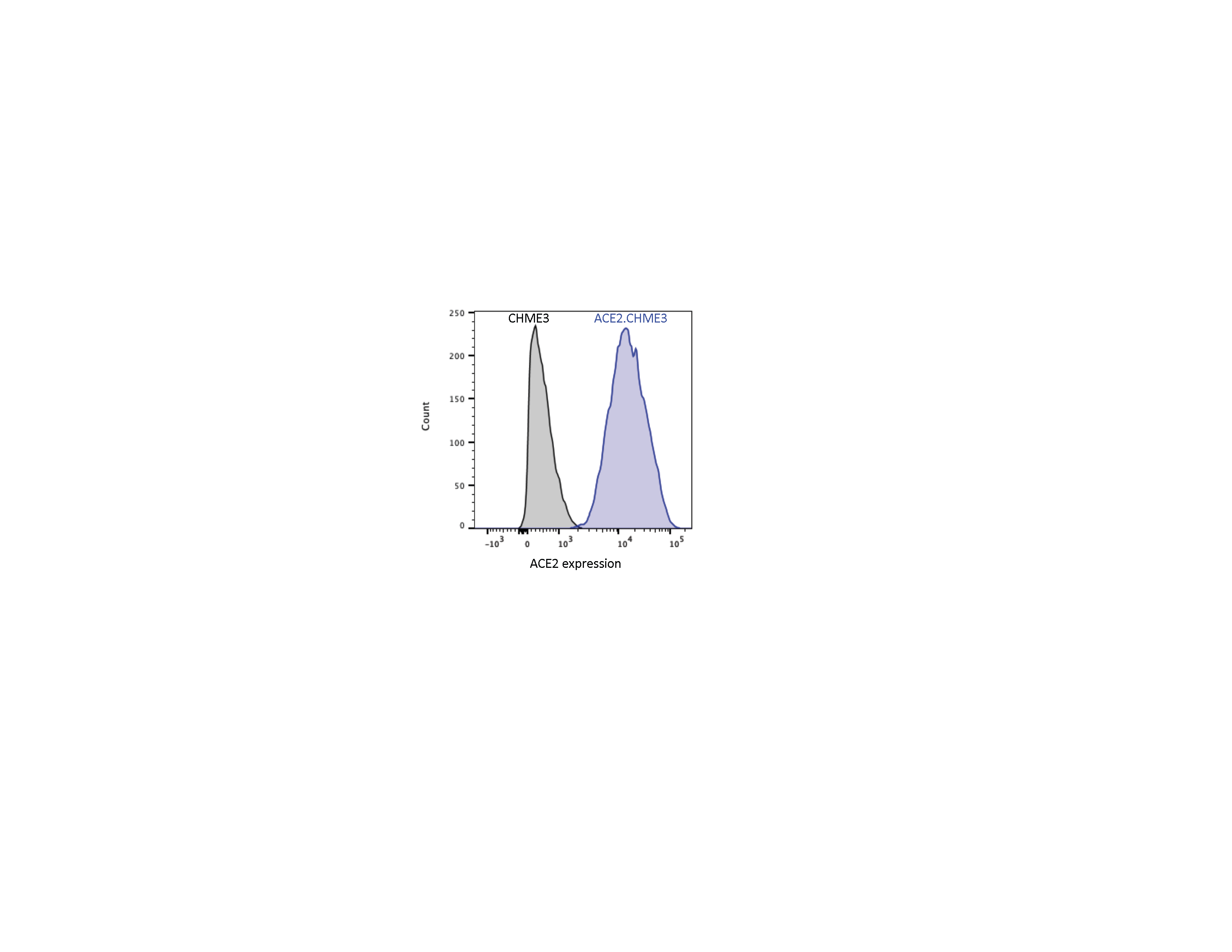

Supplement: Fig. S1 — ACE2 expression levels in ACE2.CHME3 cell line. [file mbio.01090-23-s0001.tif]

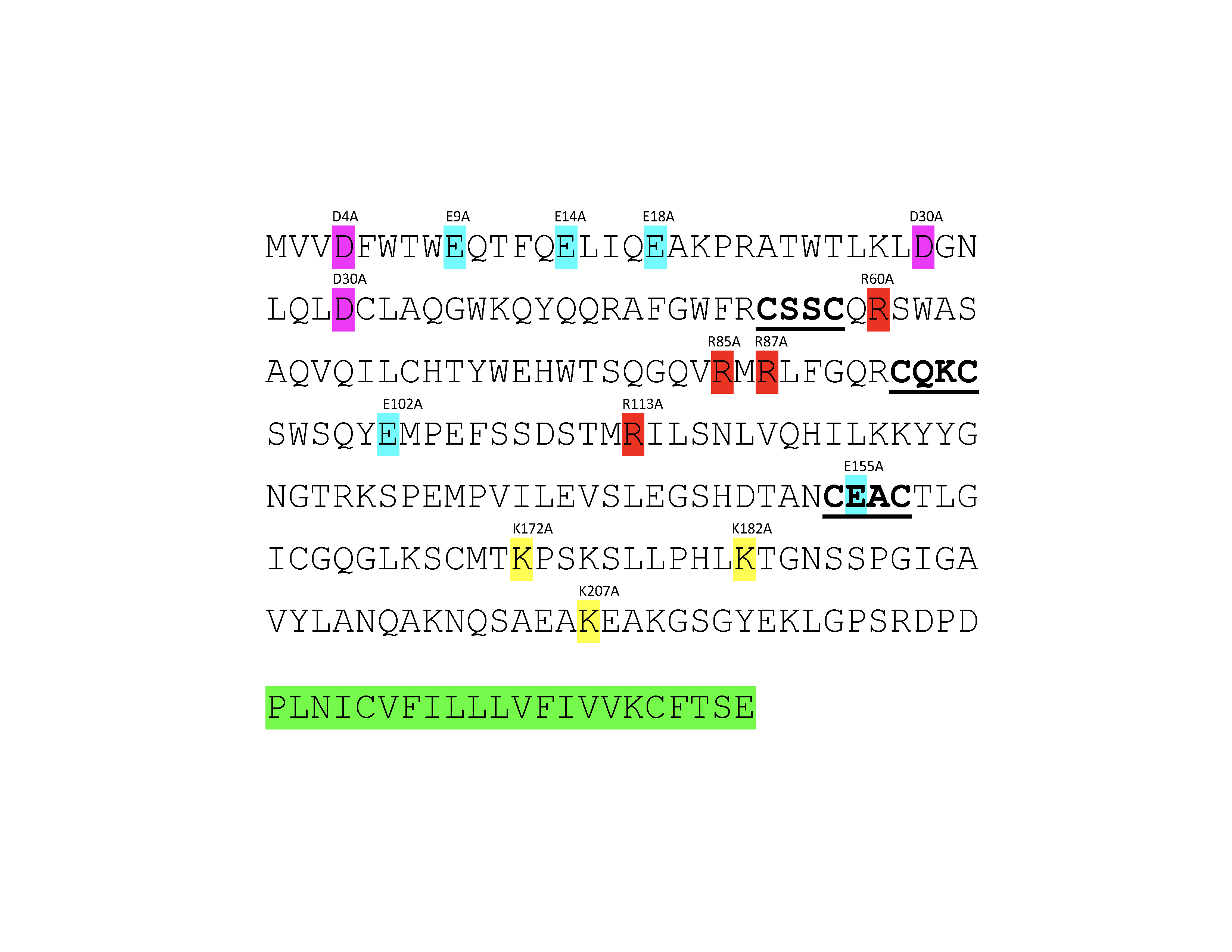

Supplement: Fig. S2 — An amino acid sequence of hRTP4 with details on the positions of single-site point mutants, truncation and ZFD mutants. [file mbio.01090-23-s0002.tif]

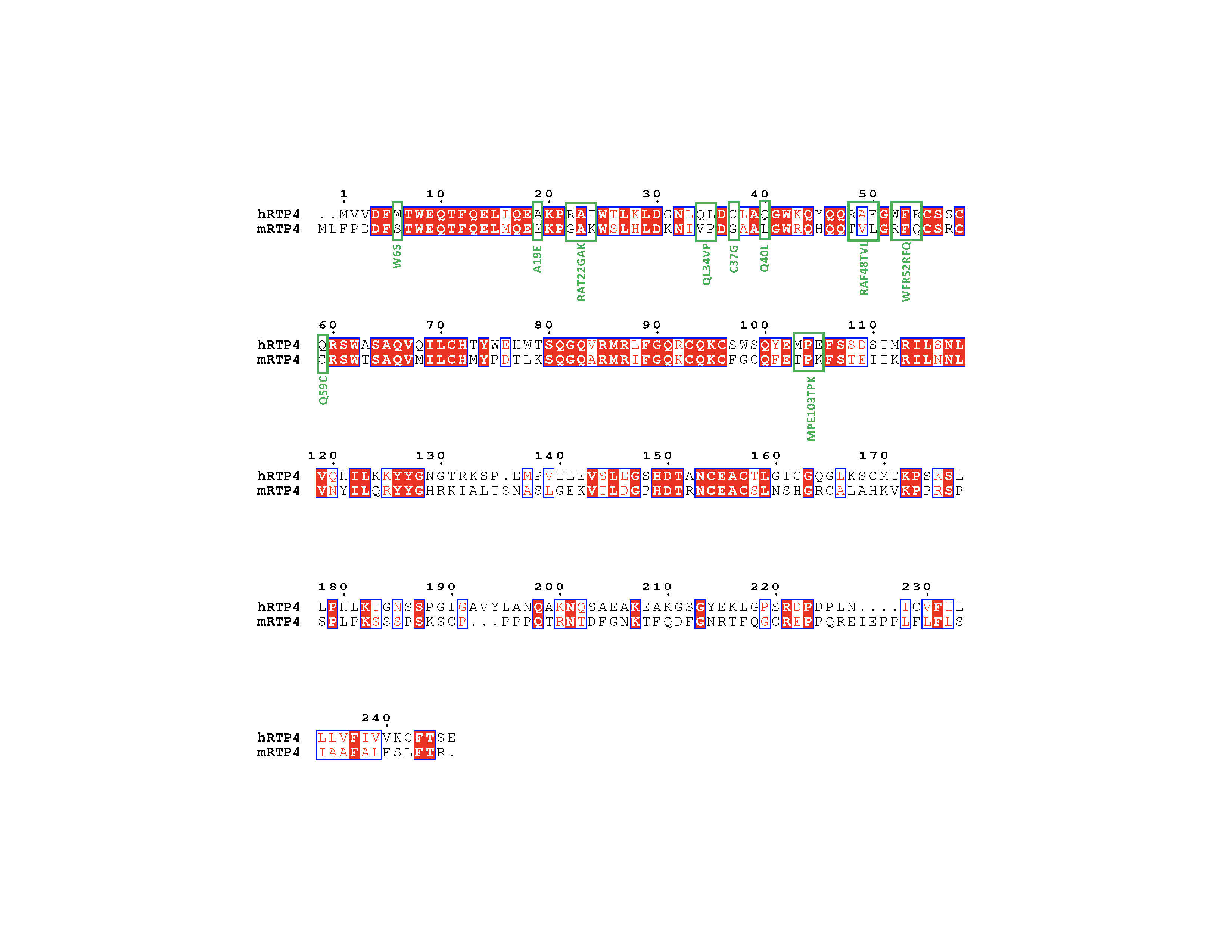

Supplement: Fig. S3 — An amino acid alignment of hRTP4 and mRTP4 with details on the human to murine RTP4 mutants. [file mbio.01090-23-s0003.tif]
