# Supplementary material for: Restriction of SARS-CoV-2 replication by receptor transporter protein 4 (RTP4)
Source: mBio. 2023 Jun 29;14(4):e01090-23. doi: 10.1128/mbio.01090-23 (PMC10470548; doi:10.1128/mbio.01090-23)
Supplement: Supplemental legends — Legends for Fig. S1 to S3 and Tables S1 to S5. [file mbio.01090-23-s0009.docx]

**Supplementary Figure legends**

**Figure S1. ACE2 expression levels in ACE2.CHME3 cell line.**

The CHME3 and ACE2.CHME3 cell lines were stained with anti-ACE2 antibody and Alexa Fluor 594-conjugated goat anti-mouse IgG secondary antibody and analyzed by flow cytometry. The experiment was performed twice with similar results.

**Figure S2. An amino acid sequence of hRTP4 with details on the positions of single-site point mutants, truncation and ZFD mutants.**

The 3CXXC ZFD are indicated in bold and underlined. The residues for 15 single-site mutations with the names of the mutations indicated above the sequences. The area shaded in green indicates the residues of TM domain truncation.

**Figure S3. An amino acid alignment of hRTP4 and mRTP4 with details on the human to murine RTP4 mutants.**

The residues for 10 human to murine RTP4 mutations with the names of the mutants indicated below the sequences and are outlined with green boxes.

**Supplementary Table legends**

**Table S1.** RNA seq data

**Table S2.** Mutagenesis strategy for 15 single-site mutants of hRTP4. Amino acid numbers shown refer to those of hRTP4. See fig S2 for the location of mutations. Aa, amino acid

**Table S3.** Mutagenesis strategy for 10 human to mouse RTP4 residues mutants. Amino acid numbers shown refer to those of hRTP4. See fig 3S for the location of mutations. Aa, amino acid

**Table S4.** Primer sequences of the plasmids constructed in the study

**Table S5.** Oligonucleotide sequences for PCR primers and probes used in the study
